# Diagnostic accuracy of severity measures of ICD-11 and DSM-5 personality disorder: clarifying the clinical landscape with the most up-to-date evidence

**DOI:** 10.3389/fpsyt.2023.1209679

**Published:** 2023-05-30

**Authors:** Luis Hualparuca-Olivera, Tomás Caycho-Rodríguez

**Affiliations:** ^1^Escuela de Psicología, Universidad Continental, Huancayo, Peru; ^2^Facultad de Psicología, Universidad Científica del Sur, Lima, Peru

**Keywords:** ICD-11, DSM-5, personality disorder, dimensional models, severity, diagnostic test accuracy

## Abstract

With the implementation of new dimensional models of personality disorder (PD) in the DSM-5 and ICD-11, several investigators have developed and evaluated the psychometric properties of measures of severity. The diagnostic accuracy of these measures, an important cross-cultural metric that falls between validity and clinical utility, remains unclear. This study aimed to analyze and synthesize the diagnostic performance of the measures designed for both models. For this purpose, searches were carried out using three databases: Scopus, PubMed, and Web of Science. Studies that presented sensitivity and specificity parameters for cut-off points were selected. There were no restrictions on the age and gender of the participants nor on the reference standard used or the settings. Study quality and synthesis were assessed using QUADAS-2 and MetaDTA software, respectively. Twelve studies were eligible covering self-reported and clinician-rated measures based on the ICD-11 and DSM-5 PD severity models. A total of 66.7% of the studies showed a risk of bias in more than 2 domains. The 10th and 12th studies provided additional metrics, resulting in a total of 21 studies for evidence synthesis. Adequate overall sensitivity and specificity (Se = 0.84, Sp = 0.69) of these measures were obtained; however, the cross-cultural performance of specific cut-off points could not be assessed due to the paucity of studies on the same measure. Evidence suggests that patient selection processes should mainly be improved (avoid case–control design), use adequate reference standards, and avoid only reporting metrics for the optimal cut-off point.

## 1. Introduction

PD is a common condition in the general population and is associated with negative outcomes for those who suffer from it and their families (1). The limited categorical conception of PD is changing towards a dimensional paradigm in current diagnostic systems (1, 2). A hybrid model is presented in the DSM-5 which combines specific categorical PD diagnoses with a dimensional Alternative Model of Personality Disorders (AMPD) to allow a smooth transition from its use to many practitioners who are accustomed to the earlier model. In the AMPD (section III of the DSM-5), criterion A is the first diagnostic step, since it allows the detection of PD (at the moderate level) and the assignment of the severity of its dysfunction from none, some, moderate, severe until extreme. Criterion B is then evaluated by assigning the maladaptive traits. In contrast, in ICD-11 the PD model is based mainly on a dimensional approach based on the severity of personality dysfunction and optionally on trait qualifiers and the borderline pattern.

Criterion A of the DSM-5 AMPD is operationalized by the Level of Personality Functioning Scale (LPFS; 3), an official measure rated by the physician to measure the patient’s personality dysfunction in four components and two domains self (identity and self-direction) and interpersonal (empathy and intimacy). Based on this measure, three semi-structured interviews have been developed: the Clinical Assessment of the Level of Personality Functioning Scale (CALF; 4), Structured Clinical Interview for the Level of Personality Functioning Scale (SCID-AMPD Module I; 5), and the Semi-Structured Interview for Personality Functioning DSM-5 (STiP 5.1; 6). Nine self-report measures have also been developed, such as the DSM-5 Levels of Personality Functioning Questionnaire (DLOPFQ; 7), and its short form (DLOPFQ-SF; 8), the Level of Personality Functioning Scale – Self-Report (LPFS-SR; 9), Level of Personality Functioning Scale – Brief Form (LPFS-BF; 10) and its second version (LPFS-BF 2.0; 11), Personality Functioning Scale (PFS; 12), Self and Interpersonal Functioning Scale (SIFS; 13), Levels of Personality Functioning Questionnaire for Adolescents from 12 to 18 Years (LoPF-Q 12–18; 14), and its short form (LoPF-Q 12–18 SF; 15).

ICD-11 severity has not been presented with an official measure, but several researchers have recently begun to develop them as CDDG guidelines for PD and related traits have been generated. The first measure developed was the Standardized Assessment of Severity of Personality Disorder (SASPD; 16) which was designed even before the final version of the guidelines was published. Other recent measures include the ICD-11 Personality Disorder Severity Scale (PDS-ICD-11; 17), Clark et al. scales (18), and PF scale of the Integrative Dimensional Personality Inventory for ICD-11 (IDPI-11; 19). Unlike criterion A of the DSM-5 AMPD, these measures have a unifactorial nature since self and interpersonal functioning are defined in a more interconnected way and linked to real-life consequences at moderate to severe levels, such as self-harm or harm to others and the reality test (20).

Diagnostic accuracy studies evaluate the performance of clinical tests (diagnostic tests), in terms of their ability to differentiate between individuals with and without the target condition, either with explanatory scientific objectives or with a pragmatic approach in clinical practice. This is done primarily through statistical analyses (e.g., sensitivity and specificity) that allow inferences to be drawn about the accuracy of clinical tests (21). Specifically, clinical tests are procedures for evaluating an individual’s current health status or predicting their future health status; and diagnostic accuracy studies provide evidence of tests for the diagnosis, staging, detection, monitoring, and surveillance of diseases (22). Improving the accuracy of the tests makes it possible for relevant referrals (or derivations) to be made, and given certain therapies to the correct patients. The clinical utility and validity of a model/measure are overlapping concepts (23) and diagnostic accuracy or precision is located differentially from the other metrics in this overlap.

Many validation studies of PD severity measures from the DSM-5 AMPD and ICD-11 models have included complementary diagnostic accuracy analyses. These studies have mainly focused on the internal structure and convergent validity of these measures, and the few studies that have made efforts to assess the precision of these measures have probably either performed them incorrectly or drawn imprecise inferences from limited methodology. Overcoming the arbitrary division into individuals with and without the disorder and exploiting the multiple gradations of severity – to improve the psychometric properties of measures of severity (1) – involves evaluating the sensitivity and specificity of each PD dysfunction threshold (target condition).

## 2. The current review

Reviews of studies on the accuracy of a diagnostic test aim to address the need for health decision makers to have access to relevant, up-to-date and high-quality information on the use of a diagnostic test as a tool for a specific setting (24). Several reviews have focused on analyzing the reliability, validity, and usefulness of PD severity measures based on the DSM-5 AMPD and ICD-11 models without delving into aspects of their diagnostic performance. Therefore, the current review aimed to determine the diagnostic accuracy of these measurements; since summarizing the literature published to date is necessary to make recommendations for clinical practice and to improve future research will be carried out. The research question was as follows: can the ICD-11 and DSM-5 severity measures be accurate for the detection of personality disorder in the general population?

We searched the literature systematically in three main databases Scopus, PubMed and Web of Science, without any language restriction by combining the following text strings: personality AND (disorder* OR patholog*) | dimension* | function* OR severi* | validity OR diagnos* OR assessment | ICD OR International Classification of Diseases | DSM-5 OR Diagnostic and Statistical Manual of Mental Disorders. The review was performed according to PRISMA-DTA (21, 25, 26) and Cochrane Handbook for Systematic Reviews of Diagnostic Test Accuracy-Version 2 (27). The search returned 531 results (2,625 in Scopus, 64 in Web of Science, and 91 in PubMed). There were no restrictions on the age and gender of the participants or for the reference standard used or the settings; because we assumed that the literature collected could be scarce. Only studies that presented sensitivity and specificity indices for one or more PD dysfunction thresholds in both models were included. The assessment of the risk of bias of the included studies was carried out using QUADAS-2 (28) and synthesis with MetaDTA v. 2.01 (29).

## 3. Results

### 3.1. Characteristics of included studies

Table 1 describes the 12 studies that represent evidence based on the subject over the last 10 years. The severity measures used in these studies include the PDS-ICD-11 and SASPD from the ICD-11 PD model; and the SIFS, LPFS-SR, LoPF-Q 12–18, LoPF-Q 12–18 SF, LPFS and algorithms of Criterion A from the PD model of the DSM-5 AMPD. These studies comprised measures administered in 12 countries (including 2 non-Western nations) and six languages. Eight of these studies used mixed samples – clinical and community – (14, 15, 30–35), and four studies used clinical samples (16, 36–38). Data from 8,390 participants were analyzed. On average, 55.9% were women; and the average age of adult and adolescent participants was 36.4 and 14.7, respectively. Study 4 (15) used data from study 7 (14); and study 12 (37), data from study 11 (36). For study 8 (34); although the target condition was initially ICD-11 PD severity, a measure designed to measure PD dysfunction according to the DSM-5 AMPD severity model was used; thus, we assigned the target condition to this last model.

**Table 1:** Description of included studies.

Study	Index test	*n* (with / without target condition)	Gender, % female	Age, M	Target condition	Reference standard	Optimal cut off point	Se	Sp	AUC [95% CI]	Administrator; informant	Population	Setting	Country	Language	Way of presenting results / other findings
1. Gutiérrez et al. (30)	PDS-ICD-11	726 (290/436)	57.4%	41.2_clinical_ / 46.3_community_	ICD-11 severity	Membership of the clinical or community group	≥8	0.80	0.73	0.84	Clinician; Self	Mixed	Outpatient mental health units / Universities	ES	Spanish	Criterion validity
2. Gutiérrez et al. (31)	SASPD	3,319 (797/2522)	61.9%	39.8_clinical_ / 41.7_community_	ICD-11 severity	Membership of the clinical or community group	≥7	0.66	0.68	0.72	Clinician; Self	Mixed	Outpatient mental health units / Universities	ES	Spanish	Criterion validity
3. Olajide et al. (29)	SASPD	110 (69/41)	54.6%	≈37	ICD-11 severity	Clinical judgment based on ICD-11 PD	≥8	0.72	0.90	0.86	Clinician; interviewer	Clinical	Hospital wards and outpatient clinics	UK, NZ	English	Diagnostic performance / cut-off point for moderate PD = 10 (se = 0.75, sp. = 0.79)
4. Zimmermann et al. (28)	LoPF-Q 12–18 SF	433 (96/ 337)	NR	NR	DSM-5 severity	SCID–II, K-DIPS _clinical_ / BPFSC-11_community_	≥36	0.80	0.88	0.92	Clinician; self and interviewer	Mixed	Inpatient and outpatient units / Public schools	CH, AT, DE	German	clinical utility / cutoff point ≥163 in community settings and ≥ 180 (se = 0.81, sp. = 0.83) in clinical settings (se = 0.75, sp. = 0.59)
5. Kerr et al. (32)	LoPF-Q 12–18	302 (94/ 298)	54.4% _clinical_ / 58.5% _community_	14.4_clinical_ / 13.1_community_	DSM-5 severity	Membership of the clinical group / BPM_community_	≥177.5	0.75	0.75	0.83	Clinician; self and informant	Mixed	Outpatient Units / Schools and youth programs	US	English	Clinical utility / cut-off point ≥176.5 if the reference test is the BPFSC-11
6. Cosgun et al. (33)	LoPF-Q 12–18	334 (52 /282	NR_clinical_ / 54.6%_community_	16.2_clinica_ / 13.5_community_	DSM-5 severity	SCID-II_clinical_ / Membership of the community group	≥176	0.84	0.68	0.79	Clinician; self and interviewer	Mixed	Psychiatric clinics / Middle and high schools	TR	Turkish	Discriminant validity (to facilitate diagnostic decisions)
7. Goth et al. (31)	LoPF-Q 12–18	433 (96 / 337)	68.7%_clinical_ / 40.2% _community_	15.4 _clinical_ / 15.7 _community_	DSM-5 severity	SCID–II, K-DIPS _clinical_ / BPFSC-11_community_	≥163	0.81	0.84	0.92	Clinician; self and interviewer	Mixed	Inpatient and outpatient units / Public schools	CH, AT, DE	German	Clinical utility
8. Gamache et al. (34)	SIFS	2,241 (778/1463)	84.6%	31.43	DSM-5 severity	Membership in clinical and community groups	≥1.30	0.79	0.86	0.90	Clinician; self	Mixed	Outpatient units of various levels of care / Online recruitment	CA	English	Delineation between participants with vs. without PD / Difficulty, moderate and severe thresholds of PD are reported with LCA
9. Hemmati et al. (35)	LPFS-SR	313 (142/171)	16.2% _clinical_ / 52.4% _community_	28.2 _clinical_ / 24 _community_	DSM-5 severity	Structured interviews based on Section II of the DSM-5 PD (outside the study)	≥306.11	0.81	0.74	0.85	Clinician; self and interviewer	Mixed	Inpatient mental health units / University	IQ	Persian	Discriminant capacity
10. Christensen et al. (38)	LPFS (SCID-5-AMPD Module I) / Criterion A algorithms	275 (192_PD_/83) / 275 (71_BPD_/204); 275 (80_AVPD_/195); 275 (30_ASPD_/245); 275 (21_OCPD_/254)	64.5%	33	DSM-5 severity	Clinical judgment based on any PD of DSM IV	≥1.5 / any two of central components	0.79_PD_ / 0.99_BPD_; 0.93_AVPD_; 0.83_ASPD_; 0.91_OCPD_	0.70_PD_ / 0.36_BPD_; 0.35_AVPD_; 0.30_ASPD_; 0.29_OCPD_	0.84 /NR	Clinician; interviewer	Clinical	Outpatient, inpatient, group psychotherapy, and substance abuse units	NO	Norwegian	Precision
11. Morey et al. (36)	LPFS	337 (248/89)	57%	39	DSM-5 severity	Clinical judgment based on any PD of DSM IV	≥2 (Moderate)	0.85	0.73	0.83	Clinician; informant	Clinical	Outpatient, inpatient, forensic, general medicine units	US	English	Relationship to existing diagnosis of PD / Little or no one (se = 1, sp. = 0); Some (se = 0.99, sp. = 0.15); Severe (se = 0.52, sp. = 0.93); Extreme (se = 0.79, sp. = 0.98)
12. Morey and Skodol (37)	Criterion A algorithms	337 (99_BPD_ /238); 337 (67_AVPD_ /270); 337 (22_OCPD_ /315); 337 (28_ASPD_ /309); 337 (35_NPD_ /302); 337 (24_STPD_ /313)	57%	39	DSM-5 severity	Clinical judgment based on BPD, AVPD, OCPD, ASPD, NPD and STPD of DSM-IV	Any two of central components	0.92_BPD_; 0.96_AVPD_; 0.80_OCPD_; 0.66_ASPD_; 0.90_NPD_; 0.87_STPD_	0.58_BPD_; 0.57_AVPD_; 0.81_OCPD_; 0.85_ASPD_; 0.66_NPD_; 0.43_STPD_	NR	Clinician; informant	Clinical	Outpatient, inpatient, forensic, general medicine units	US	English	Relationship with existing diagnosis of PD

*n*, sample size; NR, not reported; Se, sensitivity; Sp, specificity; AUC, area under curve; LCA, latent class analysis; PD, personality disorder; BPD, borderline personality disorder; AVPD, avoidant personality disorder; OCPD, obsessive compulsive personality disorder; ASPD, antisocial personality disorder; NPD, narcissistic personality disorder; STPD, schizotypal personality disorder. AT, Austria; CA, Canada; CH, Switzerland; DE, Germany; ES, Spain; IQ, Iraq; NO, Norway; NZ, New Zeeland; TR, Turkey; UK, United Kingdom; US, United States. SCID-II, structured clinical interview for the DSM-IV axis II; K-DIPS, Kinder-Diagnostic interview for mental disorders in the childhood and adolescence; BPFSC-11, borderline personality features scale for children-11; BPM, brief problem monitor.

Most studies that used mixed samples (case–control design) reported diagnostic accuracy metrics such as clinical utility statistics or discriminant or criterion validity. Only two studies reported these metrics as performance statistics and diagnostic accuracy (16, 38). Likewise, the third (16) and eleventh (36) studies reported sensitivity and specificity metrics for two or more PD dysfunction thresholds; on the other hand, study 8 (34) reported other dysfunction thresholds without these metrics. The fourth (15) and fifth (32) studies reported the optimal cut-off points and their diagnostic accuracy metrics according to the setting and reference standard, respectively. Only study 10 (38) reported additional sensitivity and specificity metrics for all cut-off points of the measure used as an index test. Finally, seven studies reported participant recruitment that reflected the dimensional spectrum of PD – e.g., students, outpatients, and hospitalized patients – (14, 16, 34, 36–38).

### 3.2. Results of the review

Due to the unanalyzed/unreported data in the reviewed studies, the diagnostic accuracy metrics provided in this section focus on the mild and moderate PD dysfunction thresholds of the ICD-11 and DSM-5 AMPD severity models, respectively. The sensitivity of the PDS-ICD-11 in the Spanish study was 0.80 and the specificity was 0.73. In the same way, a sensitivity between 0.75 and 0.85 and a specificity between 0.68 and 0.84 were found for the LoPF-Q 12–18. For the LPFS-SR, a sensitivity of 0.81 and a specificity of 0.74 were found. In addition, the sensitivity of the “any two” criteria A algorithm for the four areas of PD dysfunction ranged from 0.64 to 0.96 and its specificity from 0.29 to 0.85. Among the studies that highlighted specificity over sensitivity were those that evaluated the SASPD, SIFS, and LoPF-Q 12–18 SF. The sensitivity of SASPD ranged from 0.66 to 0.72 and its specificity ranged from 0.68 to 0.90. Similarly, the sensitivity of the SIFS was 0.79 and its specificity 0.86; likewise, the sensitivity of the LoPF-Q 12–18 SF was 0.88 and its specificity was 0.92.

### 3.3. Quality and synthesis of studies

A total of 66.7% of the studies showed a risk of bias in more than 2 domains (see Supplementary Table S1). Three studies showed bias in one domain; likewise, no study showed bias in two domains. Four studies showed bias in three domains, and in four studies we found bias in all four domains. The highest risk of bias occurred in the index test domain (91.7%), followed by the reference standard (66.7%), patient selection (58.3%), and flow and time (41.7%). To assign “risk” in each study we decided that two or more questions had to be answered affirmatively for the first two domains of QUADAS-2; while a single affirmative answer would imply an assignment of “not clear.” Five of the 12 studies showed a risk of bias in patient selection due to the case–control design used in their methodology and recruitment possibly for convenience (30–33, 35); which triggers spectrum and selection bias that could increase the sensitivity and specificity indices (39–42). The risk in this domain was not clear in two studies (14, 15), because they only used convenience sampling.

Eight of the 12 studies showed a risk of bias in the index test because there was no blinding of the results of the reference standard when applying the index test (14, 15, 30–35), generating a possible information bias that could overestimate the diagnostic performance metrics (39), and uniquely the optimal score was specified, which can also have the same effect (28). The risk in the index test was not clear for study 3 (16) due to the respective blinding, but only optimal cut-off points for mild and moderate levels of PD were reported. Eleven of the 12 studies showed bias in the reference standard because it did not correctly classify the target condition (14, 15, 30–38), causing misclassification bias or “copper standard” which can underestimate test accuracy scores (39–41). In this domain we decided to assign more weight to only one affirmative answer to assign high risk because several experts affirm that the reference standard should be the best available method to classify participants with and without the target condition (21).

**Figure 1:**
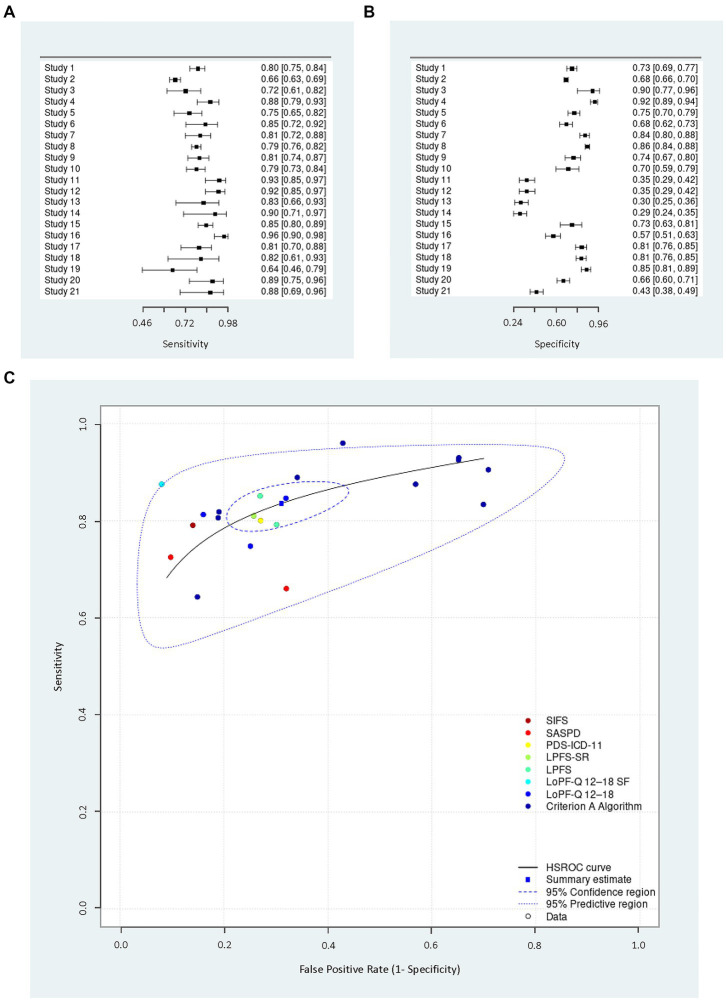
Summary plots of the reviewed studies. Panel **(A)** shows the sensitivity forest plot, panel **(B)** shows the specificity forest plot and panel **(C)** shows the HSROC plot (of random effects) by index test.

In seven of the 12 studies, bias in flow and time was noted (14, 15, 30–34), since not all people received the same reference standard, generating partial verification bias that can increase sensitivity and reduce the specificity of the test (39–41). The risk in this domain was not clear for study 9 (35) because all participants had received the same reference standard (DSM-5 Section II PD semi-structured interviews) before but outside the study. In this domain we also decided to assign more weight to only one affirmative answer to assign high bias since the “multi reference standard” in the same analysis is a common negative practice in validation studies that has a significant effect on the interpretation of the results (43). There were no applicability concerns as the review question was open-ended with no exclusion criteria for patients, reference standard, index test, or recruitment settings. Studies 10 (38) and 12 (37) contributed to further analysis, generating a total of 21 studies for the synthesis of this review (Supplementary Table S2). As seen in Figure 1, the diagnostic accuracy metrics were individually appropriate for each study; which was also demonstrated in the HSROC plot. The statisticians. Se = 0.84, Sp = 0.69, FP rate = 0.31, logit(Se) = 1.6, logit(Sp) = 0.8 supports this assertion. Specific cut-off points could not be evaluated for each of the measures because of the insufficient number of studies. Supplementary Figure S2 shows the HSROC of studies with the QUADAS-2 domains.

## 4. Discussion

Many researchers and users of the DSM-5 and ICD-11 enthusiastically welcome the transition to a dimensional approach that is more valid, reliable, and useful for the evaluation and treatment of PD than the previous diagnostic systems (44, 45). Slight variations in the conceptualization of PD in the DSM-5 10 years ago have inspired a more radical change during the preliminary versions until the final version of the ICD-11 last year (45, 46). The severity of PD dysfunction is and will be the main requirement or decision tool in both models to define who will or will not receive treatment based on a known prognosis, how many professionals to hire, and how to manage health resources (47); at the same time, clinical and community actors are educated with a recuperative and preventive vision of PD instead of stigmatizing it (48). Therefore, it is important to precisely define whether the requirements in each of the thresholds of the PD (dys)functioning continuum are adequate for its diagnosis. This review is the first to delve into the diagnostic accuracy metrics reported by studies on PD severity measures of both diagnostic systems.

Much has been said about the good psychometric levels found in severity measures (1, 2, 49); however, in this review we have found fundamental errors in the methodology that impact the analyses of diagnostic accuracy. These errors include lack of blinding when applying the index test, uniquely reporting of optimal cut-off points, imperfect reference standards, case–control design, convenience sampling, and the application of multiple reference standards in the same analysis. This, in addition to the scarcity of studies, prevents us from providing cut-off points for each of the severity measures proposed for both models. Although we would have liked to find diagnostic accuracy literature of sufficient quality for this initial objective, the reviewed studies only allow us to offer a promising general mapping of the diagnostic performance of each of these DSM-5 AMPD severity measures and ICD-11. Consequently, this study corresponds to a scoping review, allowing us to warn that inappropriate practices in the design, methodology, analysis and reporting of results on the parameters of sensitivity and specificity are avoided.

Several of the reviewed studies only reported metrics to detect the presence or absence of PD – i.e., moderate and mild levels in DSM-5 and ICD-11, respectively – ; however, they did not explore the remaining spectrum of this condition or the subclinical threshold. We were also able to observe the confusion generated by the use of terms such as “criterion validity,” “discriminant validity,” “clinical utility” among others when diagnostic accuracy metrics were used. Therefore, we recommend that the sensitivity and specificity metrics are not used to assess the differential capacity of the measure with a case–control design. Often scientific hypotheses are valid for strengthening the concepts and statements – commonly applied in preclinical studies – (50). We better positioned diagnostic accuracy metrics as quantitative analyses of clinical utility (23, 46). This includes the use of large multicenter samples with suspected PD in a given setting who are administered the index test and the same ideal reference standard for the target condition in the same study. Only by following a rigorous methodology we can truly affirm that certain cut-off points are appropriate for decision-making in the care of patients with suspected PD. Perhaps these findings suggest considering more the use of projective tests such as the Rorschach or Thematic Apperception Test (TAT), which are currently underutilized in favor of easier-to-administer tools such as questionnaires.

## 5. Final observations

The diagnostic accuracy of a test includes a set of metrics that serve as a decision tool for healthcare professionals in assigning treatment to correct patients. Since the introduction of the dimensional approach to PD in current diagnostic systems, sensitivity and specificity indices have been reported for severity measures for this condition. In this paper we attempted to summarize these metrics through the reviewed studies; however, we found substantial deficiencies in their design that prevented us from achieving this objective. Despite these limitations, this study serves as a precedent to improve our methods if we want the PD severity measures of the DSM-5 AMPD and ICD-11 to really serve what they were created for.

## Author contributions

All authors listed have made a substantial, direct, and intellectual contribution to the work and approved it for publication.

## Funding

The subsidy for the processing payment of this article was covered by Continental University (Company name: Universidad Continental S.A.C; RUC: 20319363221; Address: Av. San Carlos No. 1980, Huancayo, Peru) once accepted.

## Conflict of interest

The authors declare that the research was conducted in the absence of any commercial or financial relationships that could be construed as a potential conflict of interest.

## Publisher’s note

All claims expressed in this article are solely those of the authors and do not necessarily represent those of their affiliated organizations, or those of the publisher, the editors and the reviewers. Any product that may be evaluated in this article, or claim that may be made by its manufacturer, is not guaranteed or endorsed by the publisher.
