# Supplementary material for: Diagnostic accuracy of severity measures of ICD-11 and DSM-5 personality disorder: clarifying the clinical landscape with the most up-to-date evidence
Source: Front Psychiatry. 2023 May 30;14:1209679. doi: 10.3389/fpsyt.2023.1209679 (PMC10265646; doi:10.3389/fpsyt.2023.1209679)
Supplement: Supplementary file 1 [file Data_Sheet_1.docx]

Supplementary Material

**Table S1:** Quality assessment of included studies with QUADAS-2

| Study | Index test | Risk of bias | | | | Applicability Concerns | | |
| --- | --- | --- | --- | --- | --- | --- | --- | --- |
|  |  | PS | IT | RS | FT | PS | IT | RS |
| 1. Gutiérrez et al. (2023) | PDS-ICD-11 | ☹ | ☹ | ☹ | ☹ | ☺ | ☺ | ☺ |
| 2. Gutiérrez et al. (2021) | SASPD | ☹ | ☹ | ☹ | ☹ | ☺ | ☺ | ☺ |
| 3. Olajide et al. (2018 | SASPD | ☺ | ? | ☺ | ☺ | ☺ | ☺ | ☺ |
| 4. Zimmermann et al. (2022) | LoPF-Q 12–18 SF | ? | ☹ | ☹ | ☹ | ☺ | ☺ | ☺ |
| 5. Kerr et al. (2022) | LoPF-Q 12–18 | ☹ | ☹ | ☹ | ☹ | ☺ | ☺ | ☺ |
| 6. Cosgun et al. (2021) | LoPF-Q 12–18 | ☹ | ☹ | ☹ | ☹ | ☺ | ☺ | ☺ |
| 7. Goth et al. (2018) | LoPF-Q 12–18 | ? | ☹ | ☹ | ☹ | ☺ | ☺ | ☺ |
| 8. Gamache et al. (2021) | SIFS | ☺ | ☹ | ☹ | ☹ | ☺ | ☺ | ☺ |
| 9. Hemmati et al. (2019) | LPFS-SR | ☹ | ☹ | ☹ | ? | ☺ | ☺ | ☺ |
| 10. Christensen et al. (2019) | LPFS and Criterion A Algorithms | ☺ | ☺ | ☹ | ☺ | ☺ | ☺ | ☺ |
| 11. Morey et al. (2013) | LPFS | ☺ | ☺ | ☹ | ☺ | ☺ | ☺ | ☺ |
| 12. Morey and Skodol (2013) | Criterion A Algorithms | ☺ | ☺ | ☹ | ☺ | ☺ | ☺ | ☺ |

*Note:* PS = patient selection; IT = index test; RS = reference standard; FT = flow and timing. The icons indicate: ☺ = low risk; ? = not clear; ☹ = high risk

**Table S2:** Summary of studies derived for meta-analysis

| Study | Index test | TP | FN | FP | TN | Se | Sp | rob_PS | rob_IT | rob_RS | rob_FT | ac_PS | ac_IT | ac_RS | Target condition | Reference standard |
| --- | --- | --- | --- | --- | --- | --- | --- | --- | --- | --- | --- | --- | --- | --- | --- | --- |
| 1. Gutiérrez et al. (2023) | PDS-ICD-11 | 232 | 58 | 118 | 318 | 0.80 | 0.73 | ☹ | ☹ | ☹ | ☹ | ☺ | ☺ | ☺ | ICD-11 severity | CL / CM membership |
| 2. Gutiérrez et al. (2021) | SASPD | 526 | 271 | 807 | 1715 | 0.66 | 0.68 | ☹ | ☹ | ☹ | ☹ | ☺ | ☺ | ☺ | ICD-11 severity | CL / CM membership |
| 3. Olajide et al. (2018 | SASPD | 50 | 19 | 4 | 37 | 0.72 | 0.90 | ☺ | ? | ☺ | ☺ | ☺ | ☺ | ☺ | ICD-11 severity | ICD-11 PD (Clinical judgment) |
| 4. Zimmermann et al. (2022) | LoPF-Q 12–18 SF | 84 | 12 | 27 | 310 | 0.88 | 0.92 | ? | ☹ | ☹ | ☹ | ☺ | ☺ | ☺ | DSM-5 severity | SCID–II, K-DIPS for clinical / BPFSC-11 for community |
| 5. Kerr et al. (2022) | LoPF-Q 12–18 | 71 | 24 | 75 | 224 | 0.75 | 0.75 | ☹ | ☹ | ☹ | ☹ | ☺ | ☺ | ☺ | DSM-5 severity | CL membership / BPM for community |
| 6. Cosgun et al. (2021) | LoPF-Q 12–18 | 44 | 8 | 90 | 192 | 0.85 | 0.68 | ☹ | ☹ | ☹ | ☹ | ☺ | ☺ | ☺ | DSM-5 severity | SCID-II for clinical / CM membership |
| 7. Goth et al. (2018) | LoPF-Q 12–18 | 78 | 18 | 54 | 283 | 0.81 | 0.84 | ? | ☹ | ☹ | ☹ | ☺ | ☺ | ☺ | DSM-5 severity | SCID–II, K-DIPS for clinical / BPFSC-11 for community |
| 8. Gamache et al. (2021) | SIFS | 615 | 163 | 205 | 1258 | 0.79 | 0.86 | ☺ | ☹ | ☹ | ☹ | ☺ | ☺ | ☺ | DSM-5 severity | CL membership with PD / CM membership |
| 9. Hemmati et al. (2019) | LPFS-SR | 115 | 27 | 44 | 127 | 0.81 | 0.74 | ☹ | ☹ | ☹ | ? | ☺ | ☺ | ☺ | DSM-5 severity | CL / CM membership |
| 10. Christensen et al. (2019) | LPFS | 152 | 40 | 25 | 58 | 0.79 | 0.70 | ☺ | ☺ | ☹ | ☺ | ☺ | ☺ | ☺ | DSM-5 severity | DSM-IV Any PD (Clinical judgment) |
| 11. Christensen et al. (2019) | Criterion A Algorithm | 66 | 5 | 133 | 71 | 0.93 | 0.35 | ☺ | ☺ | ☹ | ☺ | ☺ | ☺ | ☺ | DSM-5 severity | DSM-IV BPD (Clinical judgment) |
| 12. Christensen et al. (2019) | Criterion A Algorithm | 74 | 6 | 127 | 68 | 0.93 | 0.35 | ☺ | ☺ | ☹ | ☺ | ☺ | ☺ | ☺ | DSM-5 severity | DSM-IV AVPD (Clinical judgment) |
| 13. Christensen et al. (2019) | Criterion A Algorithm | 25 | 5 | 172 | 74 | 0.83 | 0.30 | ☺ | ☺ | ☹ | ☺ | ☺ | ☺ | ☺ | DSM-5 severity | DSM-IV ASPD (Clinical judgment) |
| 14. Christensen et al. (2019) | Criterion A Algorithm | 19 | 2 | 180 | 74 | 0.90 | 0.29 | ☺ | ☺ | ☹ | ☺ | ☺ | ☺ | ☺ | DSM-5 severity | DSM-IV OCPD |
| 15. Morey et al. (2013) | LPFS | 211 | 37 | 24 | 65 | 0.85 | 0.73 | ☺ | ☺ | ☹ | ☺ | ☺ | ☺ | ☺ | DSM-5 severity | DSM-IV Any PD (Clinical judgment) |
| 16. Morey & Skodol (2013) | Criterion A Algorithm | 95 | 4 | 102 | 136 | 0.96 | 0.57 | ☺ | ☺ | ☹ | ☺ | ☺ | ☺ | ☺ | DSM-5 severity | DSM-IV BPD (Clinical judgment) |
| 17. Morey & Skodol (2013) | Criterion A Algorithm | 54 | 13 | 51 | 219 | 0.81 | 0.81 | ☺ | ☺ | ☹ | ☺ | ☺ | ☺ | ☺ | DSM-5 severity | DSM-IV AVPD (Clinical judgment) |
| 18. Morey & Skodol (2013) | Criterion A Algorithm | 18 | 4 | 60 | 255 | 0.82 | 0.81 | ☺ | ☺ | ☹ | ☺ | ☺ | ☺ | ☺ | DSM-5 severity | DSM-IV OCPD (Clinical judgment) |
| 19. Morey & Skodol (2013) | Criterion A Algorithm | 18 | 10 | 46 | 263 | 0.64 | 0.85 | ☺ | ☺ | ☹ | ☺ | ☺ | ☺ | ☺ | DSM-5 severity | DSM-IV ASPD (Clinical judgment) |
| 20. Morey & Skodol (2013) | Criterion A Algorithm | 32 | 4 | 103 | 199 | 0.89 | 0.66 | ☺ | ☺ | ☹ | ☺ | ☺ | ☺ | ☺ | DSM-5 severity | DSM-IV NPD (Clinical judgment) |
| 21. Morey & Skodol (2013) Algorithm | Criterion A Algorithm | 21 | 3 | 178 | 135 | 0.88 | 0.43 | ☺ | ☺ | ☹ | ☺ | ☺ | ☺ | ☺ | DSM-5 severity | DSM-IV STPD (Clinical judgment) |

Note: TP= true positives; FN = false negatives; FP = false positives; TN = true negatives. Se = sensitivity; Sp = specificity; CL= clinical; CM = community; PD = personality disorder; BPD = borderline personality disorder; AVPD = avoidant personality disorder; OCPD = obsessive compulsive personality disorder; ASPD = antisocial personality disorder; NPD = narcissistic personality disorder; STPD = schizotypal personality disorder. SCID–II = Structured clinical interview for the DSM–IV axis II; K-DIPS = Kinder-Diagnostic interview for mental disorders in the childhood and adolescence; BPFSC-11 = Borderline Personality Features Scale for Children–11; BPM = Brief Problem Monitor; rob_PS = risk of bias - patient selection; rob_IT = index test; rob_RS = reference standard; rob_FT = flow and timing; ac_PS = applicability concerns - patient selection; ac_IT = applicability concerns - index test; ac_RS = applicability concerns - reference standard. The icons indicate: ☺ = low risk; ? = not clear; ☹ = high risk.


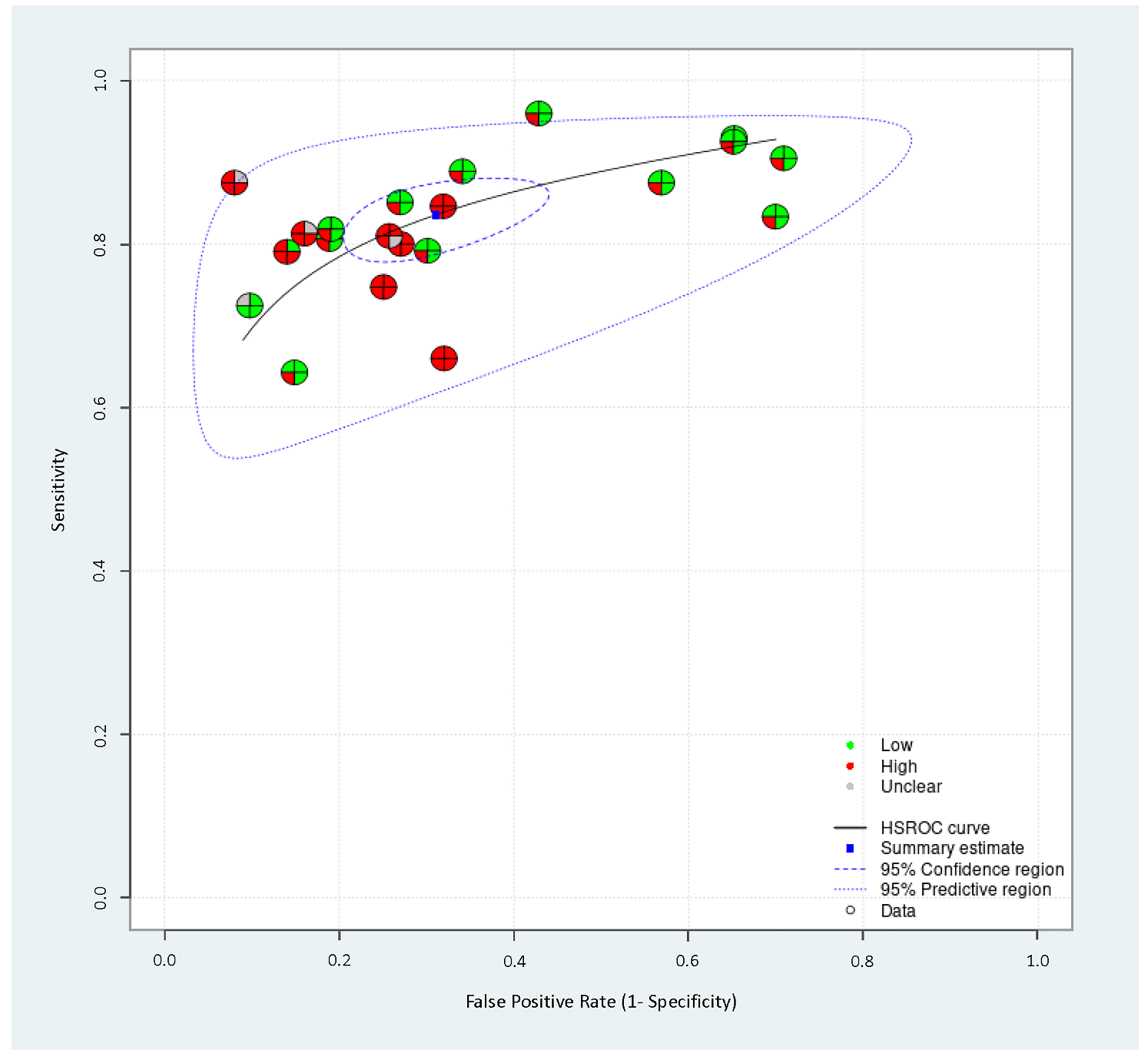


**Figure S1 | HSROC Plot of studies reviewed at risk of bias (QUADAS-2).** The index test, patient selection, reference standard, and flow and time domains are displayed in the upper left, upper right, lower right, and lower left quadrants, respectively.
